# Potential of Using Capripoxvirus Vectored Vaccines Against Arboviruses in Sheep, Goats, and Cattle

**DOI:** 10.3389/fvets.2019.00450

**Published:** 2019-12-20

**Authors:** Mahder Teffera, Shawn Babiuk

**Affiliations:** ^1^Canadian Food Inspection Agency, National Centre for Foreign Animal Disease, Winnipeg, MB, Canada; ^2^Department of Immunology, University of Manitoba, Winnipeg, MB, Canada

**Keywords:** capripoxviruses, Rift Valley fever, bluetongue, vaccine, arboviruses, vector

## Abstract

The genus capripoxvirus consists of sheeppox virus, goatpox virus, and lumpy skin disease virus, which affect sheep, goats, and cattle, respectively. Together capripoxviruses cause significant economic losses to the sheep, goat, and cattle industry where these diseases are present. These diseases have spread into previously free bordering regions most recently demonstrated with the spread of lumpy skin disease virus into the Middle East, some Eastern European countries, and Russia. This recent spread has highlighted the transboundary nature of these diseases. To control lumpy skin disease virus, live attenuated viral vaccines are used in endemic countries as well as in response to an outbreak. For sheeppox and goatpox, live attenuated viral vaccines are used in endemic countries; these diseases can also be contained through slaughter of infected animals to stamp out the disease. The thermostability, narrow host range, and ability of capripoxviruses to express a wide variety of antigens make capripoxviruses ideal vectors. The ability to immunize animals against multiple diseases simultaneously increases vaccination efficiency by decreasing the number of vaccinations required. Additionally, the use of capripoxvirus vectored vaccines allows the possibility of differentiating infected from vaccinated animals. Arboviruses such as bluetongue virus and Rift Valley fever viruses are also responsible for significant economic losses in endemic countries. In the case of Rift Valley fever virus, vaccination is not routinely practiced unless there is an outbreak making vaccination not as effective, therefore, incorporating Rift Valley fever vaccination into routine capripoxvirus vaccination would be highly beneficial. This review will discuss the potential of using capripoxvirus as a vector expressing protective arboviral antigens.

## Capripoxviruses

Capripoxviruses represent a genus of the poxviridae family under the subfamily chordopoxviriniae; the genus includes three animal virus species that have a devastating impact on sheep, goats, and cattle in Africa, Asia, and most recently Eastern Europe (1–4). The viruses in the genus are sheeppox virus (SPPV), goatpox virus (GTPV), and lumpy skin disease virus (LSDV) which affect sheep, goats, and cattle, respectively (1, 4). Capripoxviruses share 98% sequence similarity between all three species; 147 putative genes are shared between goatpox and sheeppox while lumpy skin disease virus has nine additional genes which are not functional in SPPV and GTPV (2).

It is believed that SPPV was first reported in the second century in central Asia before spreading to surrounding countries and Europe (5, 6). SPPV/GTPV are endemic in a large portion of the world [North and central Africa, the Middle East, Indian subcontinent, Southwest and central Asia (7)]. Outbreaks of sheep and goatpox can occur in new regions bordering endemic regions as illustrated by outbreaks in Mongolia and Vietnam (8). The transmission of sheep and goatpox can occur via aerosol, contact with contaminated material such as bedding, direct contact between infected animals (1, 9). Historically LSDV is a relatively new disease first described in 1929 originating in sub-Saharan Africa, where it has spread into most regions of Africa (10) and was historically thought of a disease affecting only Africa. Unfortunately, lumpy skin disease has expanded its geographic range out of Africa into the Middle East to Eastern Europe and Asia (7, 11). Most recently, LSDV spread in the Balkans including, Greece, Bulgaria, Serbia, Kosovo, Albania, Montenegro, the Caucasus Region including Russia, and Asian countries of Kazakhstan and recently China (11, 12). The rapid spread of LSDV into previously free regions is cause for concern, since if not effectively dealt with through using mass vaccination with an effective vaccine, LSDV will spread into bordering regions through either animal movement or dispersion of insect vectors (11). Therefore, there are numerous at risk countries for LSDV outbreaks in Asia with more countries becoming endemic with sheeppox, goatpox, and LSDV.

Although LSDV is not an arbovirus, insect and arthropod vectors spread the disease through mechanical transmission. The most likely vectors involved in transmission of LSDV are stable flies, mosquitos and hard ticks (13, 14). Since capripoxviruses have a tissue tropism for epithelial tissue, this allows transmission of the virus by insect or arthropod vectors to be efficient in the absence of replication in the vector (15). Capripoxviruses cause severe production losses and are world organization for animal health (OIE) listed diseases (2, 5, 16–19). LSDV also has an additional effect on lactation causing decreased milk production as well as temporary and permanent infertility (7, 19). SPPV and GTPV are associated with a relatively high morbidity and mortality (16), while LSDV is usually associated with a high morbidity and low mortality rates ranging between 1 and 5% (17, 19). The damage and loss caused by capripoxvirus on small ruminants and cattle causes substantial economic loss due to trade restrictions, limitations on movement of animals, and co-ordination and implementation of vaccination campaigns (19). This not only affects countries which rely on export of small ruminants and cattle and by products but it also impacts small scale farmers and pastoral societies whose livelihood is directly affected by the survival of their herds (5, 19). Control of sheep and goatpox can be achieved through slaughter of infected animals. Unfortunately with LSDV slaughter is not effective and can only be achieved using live attenuated vaccines (1, 2, 17); illustrated by mass vaccination of cattle in Eastern Europe where vaccination has eliminated clinical disease (6, 12).

## Currently Used Vaccines for Control of Capripoxviruses

The most effective and widely used vaccines against capripoxviruses are live attenuated vaccines (19). These live attenuated vaccines are generated by passaging field isolated viruses serially in tissue culture and/or eggs until attenuation is achieved (9, 20). An example of a commonly used vaccine is one developed in 1997 by Precausta et al. (20) which is a Romanian SPPV vaccine developed through passaging in lamb kidney cells 30 times until attenuated. This vaccine demonstrated protection against disease and generation of neutralizing serum antibodies (9, 20). The vaccine is a freeze-dried vaccine without an adjuvant and can be stored for 2 years at 6 degrees allowing for flexibility in storage and production (9). There are numerous live attenuated capripoxvirus vaccines which are used in the field reviewed by Tuppurainen et al. (21). The close antigenic relation between sheeppox, goatpox, and lumpy skin disease in theory allows a single vaccine to protect against all members. However, sheeppox virus based vaccines do not seem to protect cattle against lumpy skin disease virus. There have also been reported cases where vaccination with the RM65 strain of sheeppox virus did not elicit complete protection against LSDV (22). For this reason, capripoxvirus vaccines require evaluation in all animal species to ensure they are efficacious.

Due to regulatory issues related to trade, preventative vaccinations against capripoxviruses are not in use in disease free countries (2, 19). In South Africa, sheeppox, and goatpox vaccines are not used, instead licensed attenuated LSDV vaccines such as the OBP LSDV vaccine have been demonstrated to be safe for use and elicit long-term immunity in immunized animals (23). In other regions of Africa that are affected by all three capripoxviruses, several different capripoxvirus vaccines are used (7).

## Arboviruses

Arboviruses are a diverse group of arthropod-borne viruses that are able to replicate in arthropods and vertebrate hosts (24–26). Arboviruses are classified based on their transmission cycle and consist of a variety of RNA and DNA viruses (25). The transmission of arboviruses through arthropods occurs by an injection of an infected blood meal followed by replication of the virus in the arthropod. Viral replication occurs specifically in the salivary glands, allowing transmission to a vertebrate host; after which the infected host will most likely become viremic, a period that can last from 2 days to over a week (24–26). The ability of Arboviruses to remain in circulation is due to the maintenance of a reservoir cycle by both types of hosts (arthropod and vertebrate), which are equally necessary (24, 26). Horizontal transmission of arboviruses occurs through bites and vertical transmission through eggs (24). The main arboviral viruses affecting trade in sheep, goats, and cattle are Rift Valley fever virus and bluetongue virus.

## Bluetongue Virus

Bluetongue virus (BTV) is a virus in the family reoviridae under the genus orbivirus that causes bluetongue disease, an OIE listed hemorrhagic disease, in wild and domestic ruminants (27–30). BTV is a non-enveloped segmented double stranded RNA virus with five core proteins surrounded by a triple layered icosahedral capsid made up of two major proteins (28, 29). BTV is responsible for a significant damage of ruminant populations and the associated economic loss in countries where it is endemic (30–32). *Culicoides* midges exclusively transmit BTV to ruminants (33, 34). In sheep, clinical signs of disease are fever, nasal discharge, drooling, facial edema, and muscle weakness, accompanied by viremia (27, 35, 36). Animals surviving acute infection still remain at risk for long-term effects such as chronic dermatitis and the presence of lesions at mucosal and inter-digital surfaces (37). Mortality rates of BTV vary significantly between outbreaks; these outbreaks occur due to integration of susceptible sheep breeds into BTV endemic areas or through the spread of virus to BTV free sheep from infected sheep in areas between endemic and non-endemic areas (34). All ruminants are susceptible to BTV; however, European breeds of sheep are usually more severely affected (34). While disease is generally associated with sheep, BTV is also able to infect cattle asymptomatically; despite the disease's asymptomatic nature, IgE mediated hypersensitivity can occur in cattle (27, 38). In fact, it has been observed that insect vectors of BTV prefer to feed on cattle leading to a hypothesis that the virus reservoir is maintained by a cycle of infection going from vector to cattle (27, 39, 40).

There are 29 BTV serotypes that have been characterized to date, with different serotypes distributed among different continents, including Africa, Asia, Europe, the Americas; BTV was most recently detected in Australia in 2017 (30). There is also diversity observed within the same serotype in which viruses of a single serotype undergo genetic drift as a result of mutations and re-assortment of gene segments (30, 41, 42). Since 1988, there have been numerous BTV outbreaks in Europe which resulted in widespread vaccination campaigns to stop the spread of the disease; prior to 1988 there were only sporadic outbreaks in Mediterranean countries (41, 43, 44). Climate change is likely responsible for the rapid spread of BTV globally due to increasing vectoral capability of *Culloides* midges (45). The rapid spread of BTV and the emergence of new strains throughout the years is cause for concern and greatly impacts approaches to vaccination and surveillance. Low levels of cross-protection have been observed between different serotypes making vaccination strategies even more difficult (36).

## Vaccines Used Against Bluetongue Virus

Two types of vaccines against BTV in use are modified-live virus (MLV) vaccines or inactivated vaccines, neither of which is available for all serotypes of BTV (39, 46). MLV BTV vaccines are attenuated by passage in embryonated chicken eggs and/or tissue culture (28, 47, 48). MLV vaccines developed in South Africa are widely used in the control of BTV and its spread in Africa (38). After the re-introduction of BTV in Europe, MLV vaccines were used to vaccinate sheep despite the risks involved with re-assortment (43). Modified-live virus vaccines generally provide a good protection and are relatively inexpensive to manufacture, however, they can result in clinical signs and side effects along with the possibility of re-assortment with genes of wild type virus (38, 41). The negative effects associated with MLV vaccines include but are not limited to viremia, teratogenic effects, abortion, and reduced milk production (39). The possible unwanted effects of MLV vaccines along with trade restrictions due to the lack of differentiation between vaccinated and infected animals has highlighted the need for new vaccine strategies to control the spread of BTV (31, 39).

Inactivated or killed vaccines have also been commercially available to immunize against BTV. They are inactivated chemically, using heat, or through exposure to UV or gamma radiation (39, 49, 50). Inactivated virulent BTV strains have demonstrated long-term protective immunity (49). These vaccines have been used in Europe, namely in France and Italy (41). A downside to using inactivated virus vaccines is the decreased immunity generated due to lack of replication in these vaccines, requiring multiple injections to confer protective immunity (38). Inactivated vaccines are more expensive than using MLV vaccines, however, inactivated vaccines can prevent clinical disease, lower economic loss due to outbreaks, and allow for the safe trade of animals (36). Inactivated vaccines used against BTV serotype 8 were proven effective in Europe in 2006 during the emergence of the highly pathogenic virus by significantly reducing the potential economic impact of an outbreak (43); however they are still not considered ideal because of cost (39). Due to the obvious downsides of vaccines currently in use against BTV, it is important to consider novel vaccination strategies to account for the presence of numerous serotypes of the virus that show diverse antigenicity; a secondary issue that has yet to be addressed is the ability to distinguish vaccinated animals from infected ones.

Next generation BTV vaccines include recombinant vaccines (sub-unit, vectored, virus-like particles) and disabled infectious single cycle vaccines. Recombinant subunit vaccines use a specific protein expressed *in vitro* (28). Notably, immunization with purified VP2 resulted in the production of neutralizing antibodies and was able to protect experimentally infected sheep, demonstrating the utility of VP2 as a vaccine antigen (28, 51). VP5 can also induce neutralizing antibodies and the inclusion of both VP2 and VP5 in vaccination strategies has resulted in better protection of experimentally infected animals (52). Virus like particles (VLPs) for bluetongue have been generated by expression of VP2, VP3, VP5, and VP7 using baculovirus (53, 54). VLPs have the structural antigenicity of the virus without the genetic information, allowing these vaccines to have a high safety profile. A multi-serotype cocktail VLPs vaccine can protect against several serotypes (55).

Disabled infectious single cycle/animal (DISC/A) vaccines have also been developed against BTV. These vaccines generally lack an essential gene, which results in an inability to replicate in the host cells for more than one cycle. A DISC vaccine lacking VP6, a structural protein has been successfully produced and has been experimentally shown to provide immunity against challenge in sheep (56–58). Although a much higher dose of vaccine is required to elicit protective immunity, they are a safer alternative to using MLV vaccines.

Recombinant vectored vaccines are live attenuated virus vaccines modified to express genes encoding antigens to elicit protective immunity. Many viral vectors have a limited capacity to express foreign antigens. Therefore, it is important to select the best antigen(s) to elicit protective immunity following vaccination. For BTV, it has been shown from the use of subunit vaccines that structural proteins VP2, VP5, and VP7 confer protective immunity, VP2 being the most effective (28). Multiple different viral vectors have been generated to express different BTV proteins (VP2, VP5, VP7, NS1, and NS3) including poxviruses such as vaccinia virus (52, 59), canarypox virus (60), capripox virus (31, 61), herpes virus vectors including equine herpesvirus 1 (62), bovine herpesvirus type 4 (63), adenoviruses including canine adenovirus 2 (64), and human adenovirus 5 (65) as well as vesicular stomatitis virus (43, 66). These viral vectors elicited different levels of protection against bluetongue challenge and given the different antigens expressed as well as the different BTV challenge models it is difficult to directly compare the results. Nevertheless, there is room for improvement for vectored BTV vaccines. No viral vector to date has expressed VLPs using VP2, VP5, VP3, and VP7 proteins as previously demonstrated using baculovirus or plant expression systems (67). In addition, there is no vaccine currently available to differentiate vaccinated and infected animals (DIVA). There are currently available diagnostics for BTV serology using a competitive ELISA against VP7 (68). It may be possible to identify the specific epitope interacting with the monoclonal antibody used in the test though epitope mapping and then modify the VP7 antigen to allow for a DIVA vaccine.

## Rift Valley Fever Virus

Rift Valley fever virus (RVFV) is an enveloped segmented negative stranded RNA virus of the family Bunyaviridae, genus Phlebovirus (69, 70). It causes Rift valley fever (RVF) in livestock and humans (69, 71). Despite the presence of several lineages of RVFV, there is low genetic diversity observed with up to 99% similarity at the protein level (72).

RVFV has been responsible for devastating outbreaks throughout the African continent and has most recently been reported in the Arabian Peninsula (71, 73, 74). RVF was first described in 1931 after the infection of sheep in Kenya where close to 5,000 animals died within a month (71, 75). RVFV was endemic only in Africa and Madagascar until 2000, after when outbreaks were reported in Saudi Arabia and Yemen (73, 74). Suitable habitats for maintenance RVFV are known to be shallow depressions with the presence of wet soil or flood plains of rivers; this might explain why RVFV has only been detected in the Afrotropical region (72). Artificial interference such as irrigation and direct intervention of natural ecosystems, which modify water flow, have also been associated with increased RVFV (76). RVFV outbreaks are generally associated with increased abortion of neonates reaching 100% and mortality rates averaging 10–20% in adult livestock (71, 75). The varying mortality rate in adult ruminants is thought to be because of differences in host genetic background. Severity of RVFV can also differ within the same breed of sheep (75). Due to the zoonotic nature of RVFV, it is a threat not only to the veterinary medical communities but the overall public health of a community (71, 73, 77).

RVFV is transmitted through an infected insect bite or direct contact of infected animal tissues and body fluids (78–80). RVFV is transmitted by mosquitoes with *Aedes* spp. being the primary vectors (77, 81, 82). RVFV can also be transmitted transovarially to offspring in mosquito vectors (73, 83), allowing maintenance of the pathogen between outbreaks (83). RVFV vectors are generally divided into maintenance or amplifying, which refer to *Aedes* spp. mosquitoes found in fresh flood and semi-permanent fresh-water or *Culex* spp. found in more permanent fresh-water (72, 77). Natural events such as rainfall and flooding increase freshwater species of mosquitoes which in turn increases the risk of RVFV outbreaks in a given area (73, 84). RVFV can infect a number of vectors and vertebrate hosts including: sheep, goats, cattle, rodents, and humans (72, 84). Although mosquitoes are considered primary vectors, other vectors such as ticks (85), midges (86), and sandflies (87) have also been reported (80). Following transmission of RVFV to a host, there is an incubation period, ranging from 24 to 36 h depending on variables such as dose, strain, route of infection, and age of animal (88). The incubation period is followed by the appearance of clinical signs which can last up to 5 days usually characterized by a high fever of over 42°C and viremia (88, 89). Based on experimental infections, RVFV infections result in severe acute lethal infection, mild infection, or delayed onset complications of infection (89–91). The liver is the primary site of lesions in RVFV infections and hepatic damage is associated with severe RVF disease (73, 89, 92) although RVFV also replicates in the spleen, kidney, lung, and skin (82, 93).

Neutralizing antibodies against the RVFV proteins can protect against disease (94). Due to the damage and economic loss associated with an RVFV outbreak, successful vaccination campaigns are necessary to prevent and lower the amount of virulent RVFV circulating in endemic countries (73, 84). Unfortunately, the cyclical nature of RVFV outbreaks leads to reduced annual vaccination as the disease is out of mind.

## Vaccines Used Against Rift Valley Fever Virus

The first vaccine developed against RVFV was a live attenuated vaccine generated from the Entebbe RVFV isolate that was attenuated by serially inoculating mice interacerebrally (75, 95, 96). This vaccine, known as the Smithburn vaccine, is partially attenuated and can cause abortions and teratogenesis following vaccination. Despite this, the Smithburn vaccine and its modified live virus variants are still in use during outbreaks in non-pregnant animals (70, 72, 75, 96). Following an outbreak in Egypt in 1977, the United States army medical research institute of infectious diseases developed another RVFV vaccine known as MP12. This vaccine was generated using random mutagenesis of a virulent Egyptian strain (ZH548) of RVFV using 5-fluorouracil over twelve passages of the virus (46, 69, 94). The MP12 vaccine was more attenuated than the Smithburn vaccine as it had mutations in all its segments and showed no virulence when tested in mice. It also induced full protection in ruminants during experimental infection with a virulent RVFV strain (46, 94, 97, 98). MP12 evaluation trials in South Africa resulted in abortions and teratogenesis in pregnant ewes; despite this, MP12 is still under development to be used against RVFV in animals and humans (72, 99). The third live attenuated vaccine known as Clone 13 was obtained through a large deletion in the non-structural S protein (100, 101). Clone 13 is an ideal vaccine because of its ability to grow to very high titres in cell culture while reversion to the original strain is prevented by the large deletion in the genome. Additionally, Clone 13 was demonstrated to elicit full protective immunity in immunized animals with the lack of any negative effects in ruminants, including pregnant ewes (75, 100, 101). Despite the associated risks, live attenuated vaccines are the most effective vaccines used in the field. While most of the focus has been on the development of modified live vaccines, formalin inactivated RVFV vaccines have been used to immunize laboratory works and veterinary staff (101). The associated high cost, difficulty in production, and low yield makes inactivated RVFV vaccines not ideal in controlling the spread of RVFV in livestock (99).

Current vaccine candidates in development include recombinant RVFV vaccines, vectored subunit vaccines, subunit vaccines and virus-like particle vaccines. A recombinant MP12 vaccine has been developed where there is a mutation in the S segment similar to the clone 13 vaccine (72). Additional MP12 vaccines have been generated by deletion of the non-structural S protein completely and through a dual mutation of the non-structural S and M proteins. These vaccines were able to elicit protective immunity in trials while remaining non-virulent upon immunization (72). The other types of vaccines that have been developed are based on the expression of RVFV glycoproteins in recombinant vectors (72). The vectors that have been utilized include lumpy skin disease virus (70, 102), an alphavirus (103, 104), an adenovirus (105, 106), and the new castle disease virus (107, 108). A subunit vaccine based on Gn and Gc glycoproteins expressed using baculovirus was demonstrated to protect sheep following two vaccinations (109). Baculoviruses and tissue culture have also been utilized to express RVFV glycoproteins that then assemble into VLPs (110, 111).

## Capripoxvirus as a Vector

It has been demonstrated that other poxviruses have been used as successfully as vectors, including vaccinia virus to control rabies in wildlife (112) and fowl pox to protect chickens against Newcastle disease (113). The genomic stability, thermostability, relatively large genomic size of capripoxviruses allowing large genes to be inserted, and ability to be administered at a relatively low dose make them good candidates for use as recombinant vaccines (19, 114, 115). The tissue tropism of capripoxviruses to epithelial cells in the skin and nasal turbinate (15, 17) allows for intradermal as well as potential intranasal administration of vaccines. One of the most important features of capripoxvirus is the ability of this vector to elicit protective immunity consisting of both antibody and cellular immunity following a single immunization. This is especially important in regions that do not have high levels of veterinary services available. The major advantage of using capripoxvirus as a vector over vaccinia virus is its limited host range and being non-pathogenic to humans (23, 114, 116). This has led to the use of capripoxviruses as a suitable recombinant vector to protect cattle from diseases like rinderpest (114, 117). The thymidine kinase gene is a common gene insertion site in vectored vaccines (23). The idea of bi/multivalent vaccines is very important because it allows protective immune responses against two or more antigens of interest using a single dose of vaccine (118, 119). Due to the many advantages, capripoxviruses are increasingly being utilized as vectors to make recombinant vaccines (119, 120). Though the exact method of immunity elicited by the recombinant vaccines is not clearly defined, it is assumed to be cell mediated and humoral (117). The North African KS-1 vaccine which is a LSDV and the South African Neethling LSDV vaccine have been the most commonly used capripoxvirus vaccine strains to generate recombinant vectors (23).

The first recombinant capripoxvirus vaccine developed conferred dual protection against Rinderpest Virus (RPV) and LSDV in cattle. The recombinant vaccine was generated in lamb testicular cells using LSDV. The cells were then transfected with plasmid DNA containing the fusion (F) protein of RPV and a selectable marker (gpt) to replace the TK gene of LSDV; recombinant virus was then isolated through rounds of plaque purification (114). This vaccine was able to protect cattle completely against challenge with a virulent strain of RPV and LSDV (114, 121). The success of the first recombinant capripoxvirus experimental vaccine led to the development of numerous recombinant capripoxvirus vectored vaccines against an array of diseases afflicting small ruminants and cattle. Following the development of the first dual capripoxvirus vaccine, recombinant KS-1 capripoxvirus vaccine strains expressing either the F or hemaglutinin (H) genes of RPV were developed followed by their subsequent evaluation as possible dual vaccines against peste des petits ruminants [PPR (122)]. Both vaccines were found to be protective in experimental settings in goats against lethal challenge with PPR due to the similarity of the H and F proteins of PPR and RPV (122). In 1996, it was reported that expression of the outer capsid protein VP7 of BTV on the KS-1 strain of SPPV was able to provide partial protection of sheep against a virulent BTV challenge (31). The use of recombinant capripoxvirus to protect sheep against BTV and capripoxvirus began because of previous experiments that showed vaccination with structural proteins could elicit protective immunity in experimental animals (31). Recombinant capripoxvirus generation was also done by Ngichabe et al. (117, 118) where they generated LSDV expressing RPV H and F proteins followed by immunization. They reported full protection against challenge with both diseases; protection was also observed several years after initial vaccination in some animals (117). An attenuated LSDV vaccine strain (Neethling) was similarly utilized to successfully express a rabies virus glycoprotein in cattle where there was an antibody response from the cattle upon inoculation with the recombinant virus (115).

Wallace and Viljoen (23) generated recombinant LSDV (SA-Neethling) expressing the glycoproteins of RVFV and Bovine ephemeral fever virus (BEFV). These bivalent vaccines were constructed by inserting the foreign genes into the LSDV TK gene, conferred protective immunity against challenge with both viruses, respectively. The recombinant BEFV vaccine challenges resulted in the production of neutralizing antibodies similar to that elicited by commercial vaccines in cattle; this however, did not result in full protection in cattle while the RVFV recombinant vaccine did (23). In 2006, Diallo et al., were also able to make a recombinant capripoxvirus (KS-1 strain) expressing the H protein from PPR, they reported that at their suggested dose, it was able to protect goats against virulent PPR. This was contrary to observations where a 100X lower dose expressing the F protein of PPR showed complete protective immunity (123). The use of capripoxviruses as recombinant vectors has continued with proteins from numerous infectious viruses being expressed to provide full or partial protection against virulent challenge (124–126).

## Generation of Recombinant Capripoxvirus Vectors

### Homologous Recombination

Homologous recombination is a commonly used method of editing genomes and has been used to successfully delete or add antigen-encoding genes into capripoxviruses. Recombinant capripoxvirus vaccine generation using homologous recombination is achieved by infection of permissive cells with a capripoxvirus vector followed by a transfection with a transfer plasmid. The transfer plasmid contains selectable markers and the gene of interest with flanking regions for a non-essential capripoxvirus gene, often thymidine kinase (TK) (114–116, 120). Other insertion sites such as the IL-10 homolog gene (127) and interferon-gamma receptor-like gene have also been used as insertion sites (128). Deletion of the TK as well as open reading frames 8-18 was demonstrated to further attenuate the AV41 sheeppox vaccine (129). Deletion of the sheeppox-019 kelch like protein gene from a virulent Kazakhstan sheeppox isolate was able to attenuate the virus (130). These studies demonstrate that there are many different sites available to insert genes. There are likely many more non-essential gene targets for use as insertion sites, which have not been demonstrated to date.

**Figure 1:**
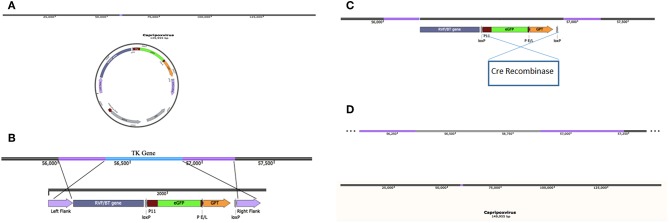
A visual representation of the generation of a capripoxvirus expressing a bluetongue virus or Rift Valley fever virus gene. **(A)** The full genome of capripoxvirus and an insertion plasmid which contains the gene of interest, two selection markers (eGFP, GPT) with loxP sites on either side, and two flanking sites corresponding to genomic regions outside the capripoxvirus gene to be replaced (e.g., Thymidine Kinase). **(B)** Alignment of the flanking regions on an insertion plasmid ideal for homologous recombination to occur with TK gene of capripoxvirus. Homologous recombination will occur in transfected cells after which selection markers can be used to identify mutant virus. **(C)** After rounds of positive selection, cre recombinase can be introduced using a plasmid or via cell lines expressing the protein to excise the selection markers present in the capripoxvirus genome. **(D)** Following successful excision of selection markers, the TK gene will have successfully been replaced with BTV/RVFV gene(s).

Wallace et al. (120) evaluated different selection methods in order to determine the most appropriate markers. The selectable markers evaluated were *Esherichia coli* (*E. coli*) β-galactosidase gene, use of green fluorescent protein (GFP) genes and/or the use of *E. coli* xanthine phosphoribosyltransferase (gpt) gene (23, 120). Efficiency wise, it is logical to use a dual selectable marker to allow for a visual confirmation and an additional marker that allows for growth in a selective media. LacZ and GFP act as visual markers where expression of these genes demonstrates homologous recombination has occurred without any further process than infection and transfection (120). Gpt is a dominant selectable marker and an added advantage as it allows for the selective growth of virus expressing the gene of interest on gpt selective media (120). Selectable markers are not acceptable to use in a licensed vaccine and can be removed in one of two ways. The first method would be to insert a P11 promoter oriented in the same direction placed before and after the selectable markers (131). The promoter is able to drive the expression of the selectable markers while also allowing for a recombinant excision of the markers once the selective pressure is removed from the growth media of the virus during negative selection (131). The second method to remove selection markers is the cre-loxP system. Similar to the presence of the P11 promoter, it involves the incorporation of a loxP sequence on either side of the selectable markers. Then following positive selection, once a pure recombinant virus is present it would be passaged in cells expressing cre recombinase which will recombine the two loxP sites and excise the selectable markers (Figure 1) (126, 132).

### CRISPR/Cas

CRISPR refers to clustered regularly interspaced short palindromic repeats. CRISPR is found in prokaryotes where it functions as a defense system to attack invading foreign DNA where the foreign DNA is inserted following the CRISPR sequence and CRISPR associated (Cas) genes to produce guide RNAs that then target the sequence of foreign DNA for destruction should it ever be re-introduced into the prokaryote (133, 134). CRISPR/Cas is a system that can be utilized in place of or in parallel with homologous recombination for the generation of recombinant vaccines. Although CRISPR/Cas has yet to be reported in the generation of recombinant capripoxvirus, it has been reported in the modification of vaccinia virus (134) and African swine fever virus (135) to improve the efficiency of genetic engineering. The similarity between pox and vaccinia opens the door for the use of CRISPR/Cas system as a gene-editing tool in the process of recombinant vaccine generation (133, 134).

### Synthetic Generation of Capripoxviruses

A novel method of recombinant poxvirus generation has recently been demonstrated involving large scale gene synthesis (136). The process involves the synthetic generation of large fragments of DNA up to 30 kb containing overlapping sequences of at least 1 kb. The fragments are synthesized in a plasmid then restricted and ligated in optimized cells with the presence of a helper virus to generate functional poxvirus (136). Using the molecular methods stated, horsepox virus was generated from 10 fragments of synthetic DNA using Shope Fibroma virus as a helper virus (136). The potential of this research is limitless in terms of new capripoxvirus vaccine generation. The ability to synthetically make capripoxvirus would allow for the modification of multiple genes at once reducing the laborious process of plaque purification and selection. In addition, using synthetic biology will allow for tailoring of the vector to enhance safety and immunogenicity.

### Recombinant Capripoxvirus Vaccines as DIVA Vaccines

Differentiating infected from vaccinated individual (DIVA) vaccines are possibly the most promising means to control and monitor the spread of rapidly spreading infectious diseases in small ruminants and cattle. Previously known as marker vaccines, DIVA vaccines refer to genetically altered conventional vaccines, which have at least one antigenic region missing (137). This results in quantifiably different antibody response from a vaccinated animal where there is a lack of antibodies against the missing antigen, allowing for the development of a test do differentiate the antibody response (16, 137, 138). This not only allows for the differentiation of vaccinated and unvaccinated animals but it also will likely decrease the amount of wildtype virus circulating in animal populations aiding in the possible eradication of a given virus (137, 138). Previously, the advantage of DIVA vaccines and accompanying serological diagnostic tests has been experimentally shown to be effective against Aujeszky's Disease virus (139) and herpes virus (138, 140). The expression of foreign proteins on capripoxvirus vectors allows for the application a DIVA companion diagnostic test allowing differentiation of vaccinated animals based on the absence of antibodies for proteins not expressed by the vectored vaccine. For example, a capripoxvirus vectored vaccine expressing the GnGc glycoproteins would generate antibodies against GnGc but would not generate antibodies against RVFV NP. The expression of foreign antigens also allows for the development of a test to detect the presence of antibodies specific to the foreign proteins expressed with the absence of other antibodies that would be present during a natural infection (141). Additionally, the simultaneous removal of a non-essential but antigenically relevant gene on the capripoxvirus vector would allow for DIVA capability for capripoxvirus vaccination with the development of an accompanying serological assay.

Currently there is no DIVA vaccine and companion diagnostic test for capripoxviruses, although there are molecular based methods available to discriminate between vaccine and wild type viruses (142–144). The development of a DIVA capripoxvirus vaccine and companion diagnostic test is theoretically feasible and technically possible. However, to do this, first a validated diagnostic ELISA is required and the antigen target used in the test must be a non-essential protein for the capripoxvirus. These two requirements are prerequisites for the development of a DIVA vaccine and companion diagnostic test.

### Future Directions to Improve Capripoxvirus Vaccine Vectors

The continuing spread of lumpy skin disease into previously free regions is leading to more countries where all capripoxvirus members are present. Since these viruses cannot be differentiated using serology, the only method to identify the specific virus is PCR and/or sequencing. The historical method used to identify the virus used the ruminant host that the virus was isolated from to characterize the virus. This worked generally well; however, there is an exception where this method did not identify the virus properly (7). The sequencing and analysis done by Tulman et al. (2, 145) has allowed for the study of capripoxvirus genes leading to studies where specific genes have been used to differentiate between sheeppox virus, goatpox virus, and lumpy skin disease virus. For example, the RPO30 and GPCR homolog genes in capripoxvirus have been used to develop real time and classical PCR tests to differentiate sheeppox from the other two capripoxviruses and between all three viruses, respectively (146–148). Although the above mentioned genes have been used to determine the species of capripoxvirus, unfortunately, the understanding of what specific genes/mutations and or gene combinations are involved in determining whether a capripoxvirus is a sheeppox, goatpox of LSDV is unknown. Analyzing the sequence information obtained from several capripoxviruses including virulent wild type and attenuated vaccines offers insight for future recombinant vaccine design (149). Understanding gene deletions found in capripoxvirus vaccines will allow strategic attenuation to target ideal virulence genes without compromising the vaccine integrity. It is likely that there are many possible gene deletion combinations available to generate a live attenuated vaccine. This information can be used to develop improved capripoxvirus vectors based on sheeppox, goatpox, and lumpy skin disease for different regions. To alleviate this issue, a universal capripoxvirus vector generated through gene synthesis, with specific gene markers for the different capripoxviruses deleted could be developed, with the inclusion of a DIVA capability with a companion diagnostic test to alleviate political issues and potentially allow the vector used in non-endemic regions. This universal capripoxvirus vaccine would be able to protect against all capripoxviruses in sheep, goats and cattle.

Capripoxvirus vectors can be tailored to include antigens for specific disease agents in the region. This is especially important in the case of BTV where there are 29 serotypes present, designing vectored vaccines based on geographically prevalent and cross-reactive serotypes is crucial to maximize the protective capability of a multivalent vaccine. The limit of the number of foreign antigens expressed simultaneously in a capripoxvirus vector is currently unknown; however, it is likely more than two antigens. In addition, it is possible that VLPs can be expressed using a capripoxvirus vector, however, this has not be demonstrated to date. It is possible to develop a capripoxvirus vector encoding the protective antigen GnGc from Rift Valley fever virus along with protective VLPs from bluetongue to generate a multivalent vaccine to protect sheep, goats and cattle from these diseases.

## Conclusions

Capripoxvirus vectors have tremendous potential for use as multivalent vaccines to protect sheep, goats and cattle from arboviruses, capripoxviruses and other devastating diseases such as peste des petits ruminants. The difficulty in vaccinating animals against arboviruses such as Rift Valley fever virus is the cyclical nature of the disease where producers do not have the resources to vaccinate for a disease that may or may not occur. Using a multivalent capripoxvirus vaccine can alleviate these issues by having a vaccine that can protect against Rift Valley fever together with endemic capripoxvirus diseases that occur much more frequently (150). The use of multivalent recombinant vaccines can provide a cost efficient strategy compared to the use of multiple conventional vaccines.

## Author Contributions

MT wrote the first draft of the manuscript. SB wrote sections of the manuscript. All authors contributed to manuscript revision, read and approved the submitted version.

### Conflict of Interest

The authors declare that the research was conducted in the absence of any commercial or financial relationships that could be construed as a potential conflict of interest.
